# Off-pump suture repair of left ventricular rupture utilizing TachoComb^®^ sheet: a case report and literature review

**DOI:** 10.1186/1749-7922-8-29

**Published:** 2013-07-27

**Authors:** Hiroki Yamaguchi, Tatsuya Nakao, Noriyuki Tokunaga, Hiromasa Nakamura, Masami Takagaki

**Affiliations:** 1 Department of Cardiovascular Surgery, New Tokyo Hospital, 1271 Wanagaya, Matsudo, Chiba 270-2232, Japan

**Keywords:** Myocardial infarction, Left ventricular rupture, Off-pump surgery, Emergency room

## Abstract

A 70-year-old woman was admitted to our institution with acute myocardial infarction. Coronary angiography demonstrated total occlusion of the left anterior descending artery, which was successfully revascularized. Four days later, the patient suddenly went into shock. The subsequent emergency operation revealed a blowout rupture of the left ventricular free wall. Several TachoComb^®^ (CSL Behring, Tokyo, Japan) sheets were applied to control bleeding and avoid the need for a cardiopulmonary bypass. Horizontal mattress sutures were used in combination with a pair of Teflon felt strips for reinforcement. The combination of TachoComb^®^ sheets and sutures is a novel hybrid method and an effective life-saving procedure for the treatment of left ventricular blowout ruptures. This approach avoids the need for a cardiopulmonary bypass. Sutureless repairs with TachoComb^®^ sheet achieve rapid hemostasis without the need for cardiopulmonary bypass. This stabilizes patient hemodynamics immediately and preserves the fragile myocardium. This allows emergency room physicians to open the chest and apply the TachoComb^®^ sheet to stabilize the patient before the cardiac surgeons arrive at the operating room. This technique will be very useful in emergency situations.

## Background

Left ventricular (LV) free wall rupture is a serious complication of acute myocardial infarction that may result in acute cardiac tamponade and sudden death. Among the various surgical procedures available for its treatment, sutureless repair using layered sheets of collagen fleece with fibrinogen-based impregnation (TachoComb^®^, CSL Behring, Tokyo, Japan) has proved the most effective [1-3]. Unlike prepare-to-use fibrin sealants, which require the coating of fibrin glue onto fleece or patching immediately before or during surgery, TachoComb^®^ is a ready-to-use fixed combination that is activated by moisture upon application, providing adherence to the resection surface. Hemostasis is generally achieved after 3–5 min of compression [4]. However, this technique alone is associated with a potential risk for future complications such as pseudoaneurysm formation and rerupture [5,6]. We therefore developed a novel hybrid method for the treatment of blowout ruptures of the LV free wall that combines TachoComb^®^ sheets with suture repair, avoiding cardiopulmonary bypass (CPB). Because this procedure can be performed without CPB, it is easily applicable even in an emergency room.

### Case presentation

A 70-year-old woman was admitted to our hospital with a 3-day-old acute myocardial infarction. Although the patient reported adherence to the prescribed medication regimen, she developed heart failure with hypotension and oliguria the next day. Coronary angiography performed under intra-aortic balloon pumping demonstrated total occlusion of the proximal left anterior descending artery (LAD). Subsequent percutaneous coronary intervention achieved successful revascularization of LAD. The patient recovered steadily and gradually. However, four days later, her condition deteriorated suddenly and she went into shock. Her echocardiography results revealed cardiac tamponade with substantial pericardial effusion. Pericardiocentesis was performed, resulting in massive continuous drainage, and she was referred to us for emergency surgery.

**Figure 1:**
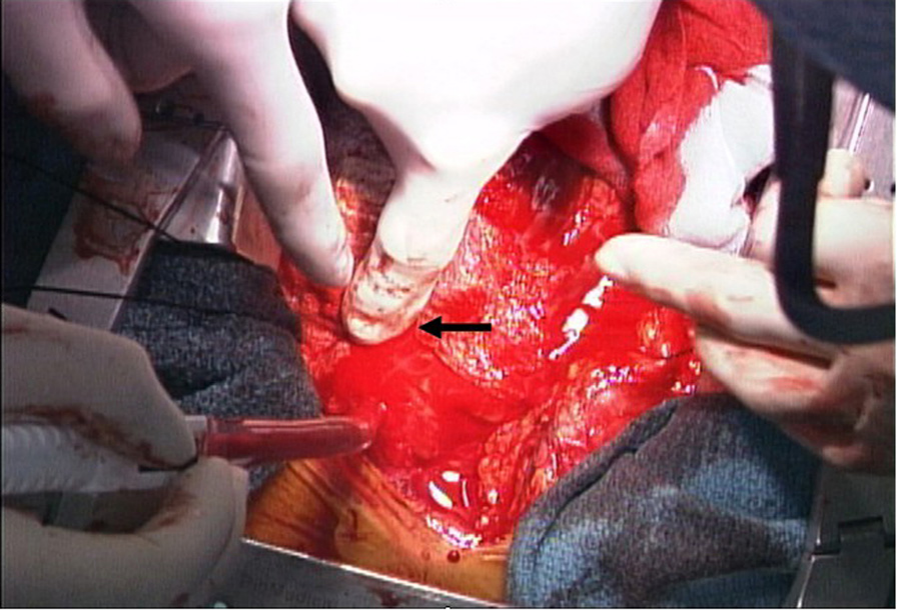
**Operative view of the ruptured left ventricle.** The major source of bleeding was a blowout rupture between the left anterior descending artery and its diagonal branch, which was controlled by manual compression (black arrow).

**Figure 2:**
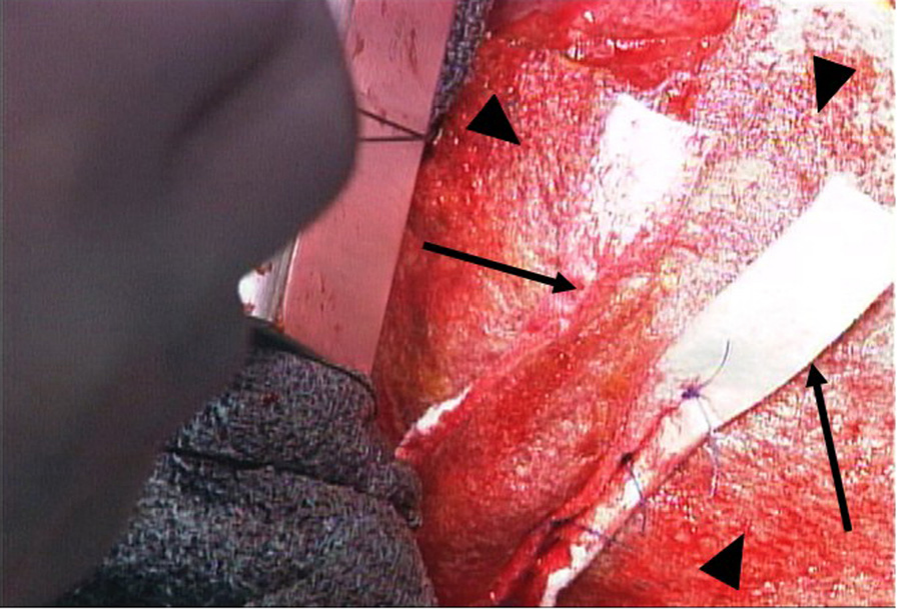
**Intraoperative view after repair.** TachoComb^®^ sheets applied to the ventricle (black arrowheads) followed by Teflon felt strip sutures (black arrows).

The patient was markedly cyanotic and in cardiogenic shock with systolic blood pressure of 70 mm Hg. A large dose of dopamine had been administered. She was intubated immediately, and the results of blood gas analysis showed marked metabolic acidosis with a pH of 7.251 and a base excess of −13.2 mmol/l. Emergency surgery was undertaken via a median sternotomy. Upon opening the pericardium, a blowout rupture of the LV free wall was found. A large volume of fresh blood was expelled rapidly from the tear at the LV base, between LAD and its diagonal branch. We were unable to measure the size of the tear, because we had to cover the area quickly with TachoComb^®^ sheets to achieve hemostasis. The LV apex was dyskinetic. A total of three TachoComb^®^ sheets (5 × 5 cm each) were applied to the bleeding point and the surrounding area of fragile necrotic tissue. The major source of bleeding was controlled, but a small amount of blood continued to flow out the lower part of the sheet (Figure 1). Four 3–0 polypropylene (SH) horizontal mattress sutures were then used to secure a pair of Teflon felt strips over the TachoComb^®^ sheets. The sutures were placed approximately 1 cm from the perforated myocardial region. Extreme care was taken to avoid LAD and its diagonal branch. After complete hemostasis was achieved, an additional TachoComb^®^ sheet and fibrin glue were applied (Figure 2). The entire LV repair was performed without CPB. The patient was transferred to the intensive care unit with dramatically improved hemodynamics. The postoperative course was uneventful, and she walked out of the hospital on day 35. The patient was followed up until 3 months, when she died because of cerebral bleeding.

### Discussion and literature review

LV free wall rupture is the third-most serious complication and the second-most common cause of death after myocardial infarction [1,7]. The patient reported herein was in an extremely serious condition on referral, and the emergency surgery performed at our institution was necessary to save her life. The new hybrid method described here was designed to control the bleeding as quickly as possible without increasing the risks for future complications such as pseudoaneurysms and reruptures [5,6].

Various procedures and strategies have been developed to treat LV free wall ruptures (Table 1). The choice among them is made on the basis of three main considerations: (1) type of rupture, (2) with or without CPB, and (3) suture closure or sutureless repair. Blowout ruptures are often treated by infarctectomy combined with suture closure and/or patch repair, usually with CPB [7-10]. Oozing/sealed ruptures are often treated by sutureless repair without CPB [1-3,10]. Recent myocardial infarction decreases the heart’s tolerance to subsequent global ischemia even when protected by hypothermic cardioplegia. Therefore, it is preferable to repair a ruptured LV free wall without CPB. Although the suture closure technique is a classic standard procedure, it is difficult to suture fragile myocardium because of the risk of mechanical tearing [1,2,11]. Many surgeons have recently reported that sutureless repair using TachoComb^®^ sheets can efficiently achieve hemostasis [3,5,6,11]. However, this strategy is not usually suitable for blowout ruptures, where the myocardial tear is often large and bleeding is copious [1-3]. Although Nishizaki et al. [11] reported successful sutureless repairs with use of the TachoComb^®^ sheet for a blowout rupture from a 1-cm tear, the risks of such an approach are possible future complications such as pseudoaneurysm and rerupture [5,6].

**Table 1:** Reference review for surgical repair of the left ventricular free wall rupture

**Reference**	**Year**	**Article type**	**No. of pts.**	**Rupture type**	**Surgical procedures**	**CPB**
Stiegel et al. [9]	1987	Case report	1	Blow-out	Suture closure and patch repair	Yes
Sutherland et al. [8]	1996	Case report/Review	1	Blow-out	Suture closure	Yes
Reardon et al. [7]	1997	Case report	1	Blow-out	Infarctectomy and patch repair	Yes
Iemura et al. [1]	2001	Original article	17	Oozing (n=14), Blow-out (n=3)	Infarctectomy and patch repair (n=1), Direct closure (n=4), Patch repair (n=4), Sutureless patch repair (n=7), Endventricular patch closure (VSP) (n=1)	Yes (n=12)
						No (n=5)
Lachapelle et al. [2]	2002	Original article	6	Oozing (n=3), Blow-out (n=3)	Sutureless patch repair (n=6)	Yes (n=4)
						No (n=2)
Fukushima et al. [5]	2003	Case report	1	Oozing	Sutureless repair with TachoComb	No
Nishizaki et al. [11]	2004	Case report	1	Blow-out	Sutureless repair with TachoComb	No
Muto et al. [3]	2005	Case report	1	Oozing	Sutureless repair with TachoComb	No
Kimura et al. [6]	2005	Case report	1	Blow-out	Sutureless repair with TachoComb	No
Sakaguchi et al. [10]	2008	Original article	32	Unknown (n=28), Blow-out(n=4)	Sutureless repair with autologous pericardial patch and gelatinresorcin formaldehyde glue +− additional sutures	Yes (n=6)
						No (n=26)
Pocar et al. [13]	2012	Original article	3	Unknown	Sutureless repair with TachoSil combined with pericardial patch and fibrin glue	Yes
Raffa et al. [14]	2013	Original article	6	Oozing (n=4), Blow-out (n=2)	Sutureless repair with TachoSil	Yes (n=3)
						No (n=3)

*No. of pts.* Number of patients, *CPB* Cardiopulmonary bypass, *VSP* Ventricular septal perforation.

The advantages of sutureless repairs with TachoComb^®^ sheets include rapid hemostasis without the need for CPB, which allows for the immediate stabilization of patient hemodynamics and preservation of the fragile myocardium [2,3,5,6]. Furthermore, even physicians in an emergency room can open the chest and apply a TachoComb^®^ sheet to stabilize the patient before the cardiac surgeons arrive at the operating room. We therefore developed a new hybrid method that combines use of the TachoComb^®^ sheet with suture closure to utilize the advantages of both procedures. Because of the risk of mechanical tearing, we do not recommend the use of this technique for tears >1 cm. However, the procedure can be performed safely without CPB, which represents a substantial advantage in emergency situations.

Although TachoComb^®^ has frequently been used for the treatment of both venous and arterial bleeding, anaphylactic reactions have been reported after the repeated use of hemostatic agents such as TachoComb^®^ that contain aprotinin. Because aprotinin is also associated with risks of renal failure, a new product, TachoSil^®^ (Nycomed, Zurich, Switzerland), which lacks aprotinin and contains human rather than bovine thrombin, has been developed. TachoSil^®^ is known to be equally hemostatic to TachoComb^®^[12]. Several cases of LV rupture have been treated successfully utilizing TachoSil^®^ (Table 1) [13,14].

Our report has some limitations. First, the report here describes a single case. Further investigation including postoperative follow-up in a large number of patients will be necessary to support our hypothesis. Second, our technique does not address LV aneurysms, which could lead to heart failure and/or thromboembolisms. TachoComb^®^ sheets covering the LV surface could complicate a concomitant or subsequent coronary artery bypass graft. Indeed, Iemura et al. [1] maintain that if subsequent coronary artery bypass grafting is needed, identification and exposure of the coronary artery will be difficult because of the widely and deeply piled collagen hemostats. However, the main goal of surgery for LV rupture is to save the patient’s life by relieving the cardiac tamponade and to close the rupture [2,3]. We believe that our method maximizes the chance of patient survival and provides a novel option for emergency room physicians.

## Conclusions

A novel hybrid method that combines TachoComb^®^ sheets with reinforcing sutures was effective in quickly achieving hemostasis without the need for CPB. This represents a substantial advantage in the context of emergency medicine.

### Consent

Written informed consent was obtained from the patient’s family for publication of this case report and any accompanying images. A copy of the written consent is available for review by the Editor-in-Chief of this journal.

## Competing interests

We declare that we have no competing interests.

## Authors’ contributions

HY performed the surgery, supervised the patient’s care, drafted the manuscript, and approved the version submitted for publication. TN, NT, and HN assisted with patient care and have been involved in drafting the manuscript. MT has been involved in drafting and revising the manuscript. All authors read and approved the final manuscript.
